# Augmentation of Cathepsin Isoforms in Diabetic db/db Mouse Kidneys Is Associated with an Increase in Renal MARCKS Expression and Proteolysis

**DOI:** 10.3390/ijms241512484

**Published:** 2023-08-05

**Authors:** Mohammed F. Gholam, Niharika Bala, Yunus E. Dogan, Abdel A. Alli

**Affiliations:** 1 Department of Physiology and Aging, University of Florida College of Medicine, Gainesville, FL 32610, USA; 2 Department of Basic Medical Sciences, College of Medicine, King Saud bin Abdulaziz University for Health Sciences, Jeddah 22384, Saudi Arabia; 3 Department of Medicine Division of Nephrology, Hypertension and Renal Transplantation, University of Florida College of Medicine, Gainesville, FL 32610, USA

**Keywords:** MARCKS, PKC, cathepsin, proteolysis, kidney

## Abstract

The expression of the myristoylated alanine-rich C-kinase substrate (MARCKS) family of proteins in the kidneys plays an important role in the regulation of the renal epithelial sodium channel (ENaC) and hence overall blood pressure regulation. The function of MARCKS is regulated by post-translational modifications including myristoylation, phosphorylation, and proteolysis. Proteases known to cleave both ENaC and MARCKS have been shown to contribute to the development of high blood pressure, or hypertension. Here, we investigated protein expression and proteolysis of MARCKS, protein expression of multiple protein kinase C (PKC) isoforms, and protein expression and activity of several different proteases in the kidneys of diabetic db/db mice compared to wild-type littermate mice. In addition, MARCKS protein expression was assessed in cultured mouse cortical collecting duct (mpkCCD) cells treated with normal glucose and high glucose concentrations. Western blot and densitometric analysis showed less abundance of the unprocessed form of MARCKS and increased expression of a proteolytically cleaved form of MARCKS in the kidneys of diabetic db/db mice compared to wild-type mice. The protein expression levels of PKC delta and PKC epsilon were increased, while cathepsin B, cathepsin S, and cathepsin D were augmented in diabetic db/db kidneys compared to those of wild-type mice. An increase in the cleaved form of MARCKS was observed in mpkCCD cells cultured in high glucose compared to normal glucose concentrations. Taken together, these results suggest that high glucose may contribute to an increase in the proteolysis of renal MARCKS, while the upregulation of the cathepsin proteolytic pathway positively correlates with increased proteolysis of MARCKS in diabetic kidneys, where PKC expression is augmented.

## 1. Introduction

Several studies have shown protein kinase C (PKC) activation in kidney cells cultured in high glucose [1,2] and in kidney tissue of diabetic animal models [3,4]. PKC activation in response to hyperglycemic conditions leads to production of the profibrotic cytokine transforming growth factor β (TGF-β1) and expansion of the extracellular matrix, which contributes to pathophysiological features of diabetic nephropathy [5]. The activation of PKC in diabetic kidneys plays an important role in pathophysiology, since PKC phosphorylates and regulates a myriad of proteins including those that regulate the actin cytoskeleton.

The myristoylated alanine-rich C-kinase substrate (MARCKS) family of proteins includes MARCKS and MARCKS-like protein 1 (MLP1); these proteins are among the most prominent substrates of PKC. In the kidneys, MARCKS and MLP1 have been shown to play an important role in maintaining the integrity of the actin cytoskeleton and regulating the function of epithelial transport mechanisms at the luminal plasma membrane. MARCKS [6,7,8] and MLP1 [9,10] both interact with epithelial sodium channels (ENaC) in renal epithelial cells. These proteins increase the density of ENaC at the membrane and its open probability by sequestering and increasing the local concentration of phosphatidylinositol 4,5-bisphosphate (PIP2) in close proximity to ENaC [6]. PIP2 stabilizes ENaC in an open confirmation at the membrane [10,11]. Phospholipase C (PLC) hydrolyses PIP2 and reduces its concentration at the membrane [12]. The interaction between MARCKS and ENaC in the kidneys is regulated by post-translational modifications, proteases, and protease inhibitors [7]. A previous study by our group showed a strong association between MARCKS and ENaC in diabetic db/db kidneys, which was attenuated by human alpha-1 antitrypsin treatment [13].

Although the regulation of MARCKS expression and function by PKC has been studied in various cells [14,15], its regulation in diabetic kidneys is not well understood. In order to address these knowledge gaps, the goals of this study were to (1) investigate the proteolysis and expression of MARCKS in diabetic db/db kidneys, (2) investigate changes in the expression and activity of multiple proteases, including different isoforms of cathepsins in diabetic kidneys, (3) investigate changes in PLC protein expression in diabetic kidneys, and (4) investigate whether acute high glucose treatment of ENaC expressing renal epithelial cells itself induces proteolysis of MARCKS.

## 2. Results

### 2.1. Increased Proteolysis of MARCKS Proteins in Diabetic db/db Kidneys

First, to confirm these animals were diabetics, we measured their blood glucose concentrations as shown in Table 1. The mean blood glucose concentration for the wild-type mice was 115.14 ± 14.84 mg/dL and for the db/db mice was 536.14 ± 12.7 mg/dL. Furthermore, urinary albumen and creatinine were comparable between the groups as shown in Table 1.

**Figure 1:**
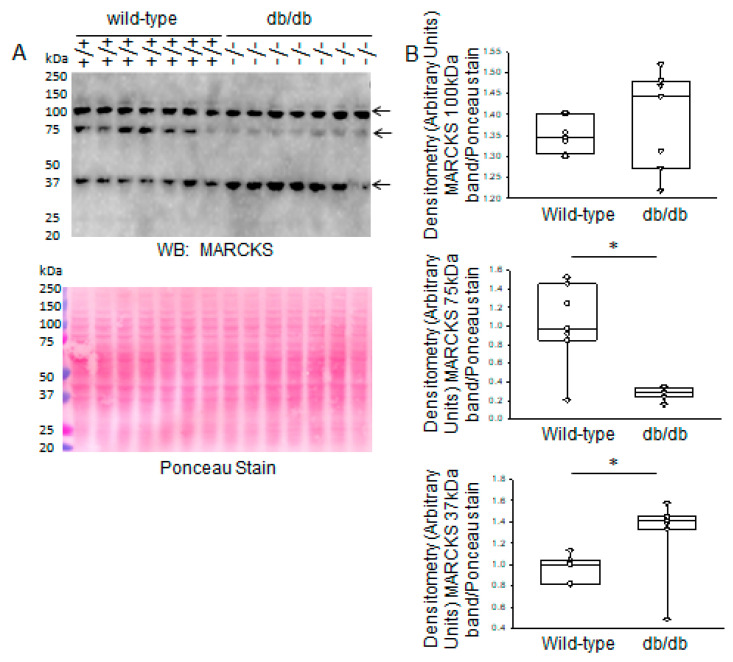
Western blot and densitometric analysis of myristoylated alanine-rich C-kinase substrate (MARCKS) protein expression in the kidneys of healthy wild-type and diabetic db/db mice. (**A**) Western blot for MARCKS protein. Arrows indicate immunoreactive bands corresponding to the uncleaved and cleaved forms of MARCKS protein. Ponceau stain was used to assess lane loading. (**B**) Densitometric analysis of the immunoreactive bands in panel A indicated by an arrow. *N* = 7 mice in each group. * represents a *p*-value of <0.05.

The subcellular localization of MARCKS is known to be regulated by phosphorylation and proteolysis. Therefore, we investigated changes in the cleaved form of MARCKS in the kidneys of diabetic db/db mice and wild-type littermate mice. There was more MARCKS proteolysis, indicated by an increase in the 37 kDa band in diabetic db/db kidneys compared to those of wild-type mice, as shown in a Western blot that was probed for various forms of MARCKS using a recombinant antibody against the MARCKS protein (Figure 1). Additionally, there was less abundance of a 75–80 kDa form of MARCKS in the diabetic db/db kidneys, while the 100 kDa immunoreactive band did not show an appreciable difference between the two groups (Figure 1).

### 2.2. Increased PKC Isoform Expression in Diabetic db/db Kidneys

**Figure 2:**
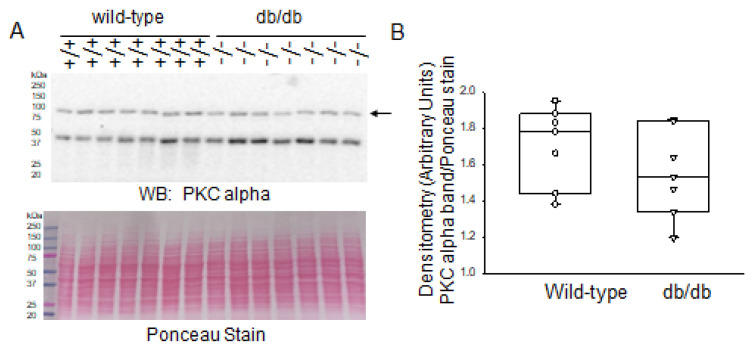
Western blot and densitometric analysis of PKC alpha protein expression in the kidneys of healthy wild-type and diabetic db/db mice. (**A**) Western blot for PKC alpha protein. Arrow indicates the immunoreactive band corresponding to PKC alpha protein. Ponceau stain was used to assess lane loading. (**B**) Densitometric analysis of the immunoreactive band in panel A indicated by an arrow. *N* = 7 mice in each group.

PKC activation in diabetic kidneys is known. For example, it has been reported that high glucose causes activation of PKC-alpha and PKC-epsilon isoforms in whole kidney lysates of a streptozotocin-induced rat model of type 1 diabetes [16]. Here, we investigated whether PKC isoforms are increased in the kidney cortex of db/db mice representing a model of type 2 diabetes. Protein expression of PKC-alpha in the kidneys did not show an appreciable difference between diabetic db/db mice and wild-type littermate mice (Figure 2).

**Figure 3:**
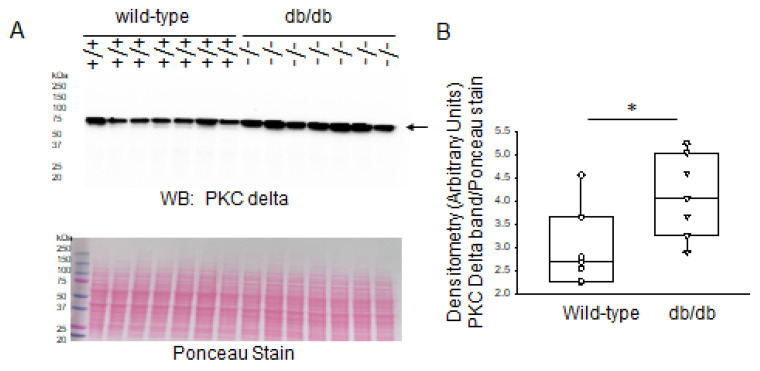
Western blot and densitometric analysis of PKC delta protein expression in the kidneys of healthy wild-type and diabetic db/db mice. (**A**) Western blot for PKC delta protein. Arrow indicates the immunoreactive band corresponding to PKC delta protein. Ponceau stain was used to assess lane loading. (**B**) Densitometric analysis of the immunoreactive band in panel A indicated by an arrow. *N* = 7 mice in each group. * represents a *p*-value of <0.05.

Next, we investigated changes in the protein expression of PKC-delta in the diabetic db/db kidneys compared to those of wild-type littermate mice. Western blot and densitometric analysis showed a greater amount of PKC-delta protein being expressed in the diabetic db/db kidneys compared to the healthy wild-type kidneys (Figure 3).

**Figure 4:**
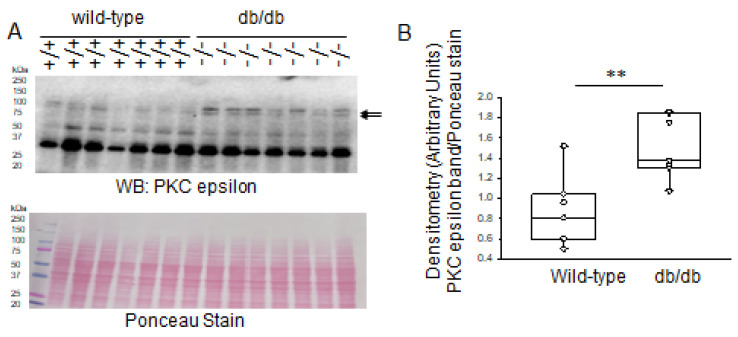
Western blot and densitometric analysis of PKC epsilon protein expression in the kidneys of healthy wild-type and diabetic db/db mice. (**A**) Western blot for PKC epsilon protein. Arrow indicates the immunoreactive band corresponding to PKC epsilon protein. Ponceau stain was used to assess lane loading. (**B**) Densitometric analysis of the immunoreactive band in panel A indicated by an arrow. *N* = 7 mice in each group. ** represents a *p*-value of <0.01.

PKC epsilon is also expressed in the mouse kidneys. Similar to PKC delta, Western blot and densitometric analysis showed greater levels of PKC epsilon protein being expressed in the kidneys of diabetic db/db mice compared to those of healthy wild-type control mice (Figure 4).

### 2.3. Expression Levels of Proteases in Diabetic db/db Kidneys Compared to Kidneys of Wild-Type Control Mice

**Figure 5:**
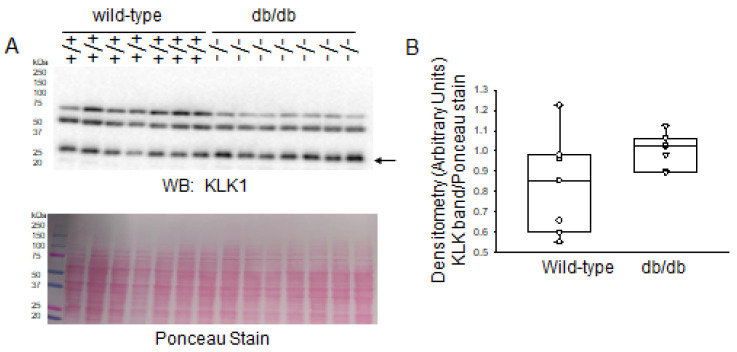
Western blot and densitometric analysis of Kallikrein 1 (KLK1) protein expression in the kidneys of healthy wild-type and diabetic db/db mice. (**A**) Western blot for KLK1 protein. Arrow indicates the immunoreactive band corresponding to kallikrein 1 protein. Ponceau stain was used to assess lane loading. (**B**) Densitometric analysis of the immunoreactive band in panel A indicated by an arrow. *N* = 7 mice in each group.

In addition to being regulated by PKC phosphorylation, the function of MARCKS is regulated by proteolysis. Our group previously investigated the role of MARCKS and ENaC proteolysis in the kidneys [8]. Calpain-1 and calpain-2 protein expression and activity in cardiac mitochondria is known to be increased in diabetes leading to myocardial injury and dysfunction [17]. The expression and activities of other proteases, including cathepsins, in diabetic kidneys compared to healthy kidneys have not been fully investigated. Here, we focused on investigating changes in expression and activity of specific proteases within the kidneys of diabetic and healthy mice that are known to cleave MARCKS and/or ENaC. First, we investigated changes in kidney kallikrein 1 (KLK1) protease expression between the two groups. Western blot and densitometric analysis did not show an appreciable change in KLK1 between the two groups (Figure 5).

**Figure 6:**
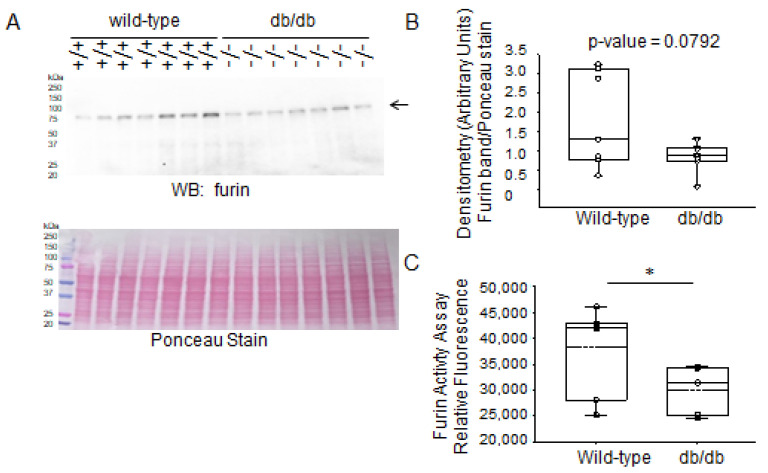
Western blot and densitometric analysis of furin protein expression in the kidneys of healthy wild-type and diabetic db/db mice. (**A**) Western blot for furin protein. Arrow indicates the immunoreactive band corresponding to furin protein. Ponceau stain was used to assess lane loading. (**B**) Densitometric analysis of the immunoreactive bands in panel A indicated by an arrow. (**C**) Furin activity (shown as relative fluorescence) in kidney lysates from diabetic db/db mice compared to wild-type mice. *N* = 7 mice in each group. * represents a *p*-value of <0.05.

Next, we investigated changes in the protein expression of furin between the two groups. There were significantly lower levels of furin activity in the diabetic db/db kidneys compared to the kidneys of wild-type mice (Figure 6).

**Figure 7:**
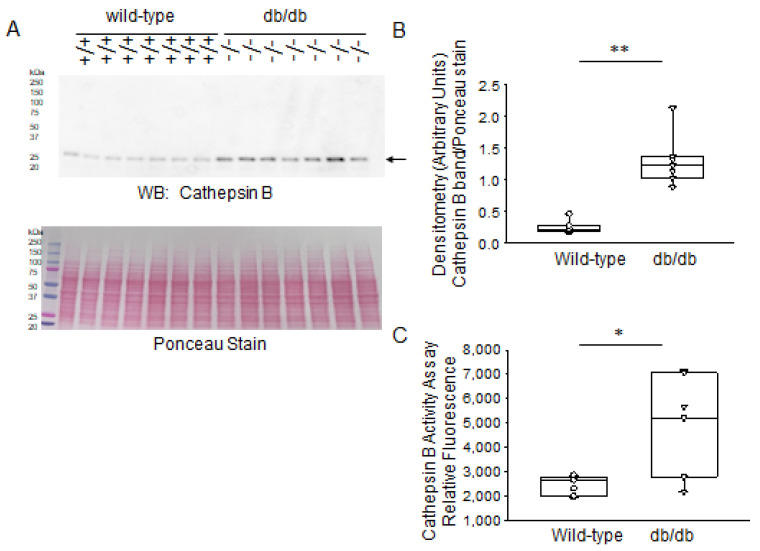
Western blot and densitometric analysis of cathepsin B protein expression in the kidneys of healthy wild-type and diabetic db/db mice. (**A**) Western blot for cathepsin B protein. Arrow indicates the immunoreactive band corresponding to cathepsin B protein. Ponceau stain was used to assess lane loading. (**B**) Densitometric analysis of the immunoreactive band in panel A indicated by an arrow. (**C**) Cathepsin B activity (shown as relative fluorescence) in kidney lysates from diabetic db/db mice compared to wild-type mice. *N* = 7 mice in each group. * represents a *p*-value of <0.05. ** represents a *p*-value of <0.01.

It is well established that MARCKS is cleaved by cathepsins. However, protein expression and activity of various members of the cathepsin family has not been investigated in diabetic db/db kidneys compared to those of healthy wild-type mice. Western blot and densitometric analysis showed greater basal levels of cathepsin B protein expression in the kidneys of diabetic db/db mice compared to the control group (Figure 7A,B). Consistent with the changes in cathepsin B protein expression, there was greater cathepsin B activity in the diabetic db/db kidneys compared to those of wild-type littermate mice (Figure 7C).

**Figure 8:**
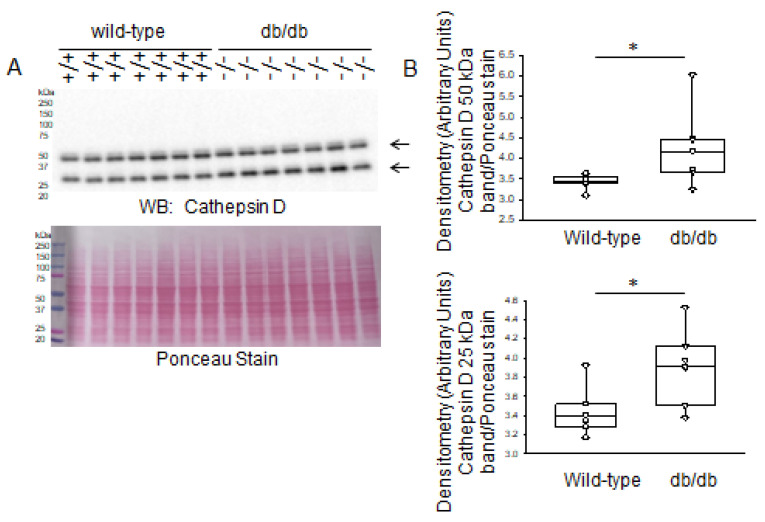
Western blot and densitometric analysis of cathepsin D protein expression in kidneys of healthy wild-type and diabetic db/db mice. (**A**) Western blot for cathepsin D protein. Arrow indicates the immunoreactive band corresponding to cathepsin D protein. Ponceau stain was used to assess lane loading. (**B**) Densitometric analysis of the immunoreactive bands in panel A indicated by an arrow. *N* = 7 mice in each group. * represents a *p*-value of <0.05.

Cathepsin D is another abundantly expressed member of the cathepsin family that is expressed in the kidneys. Similar to cathepsin B, Western blot and densitometric analysis showed greater basal levels of cathepsin D protein expression in the diabetic db/db kidneys compared to those of healthy wild-type littermate mice (Figure 8).

**Figure 9:**
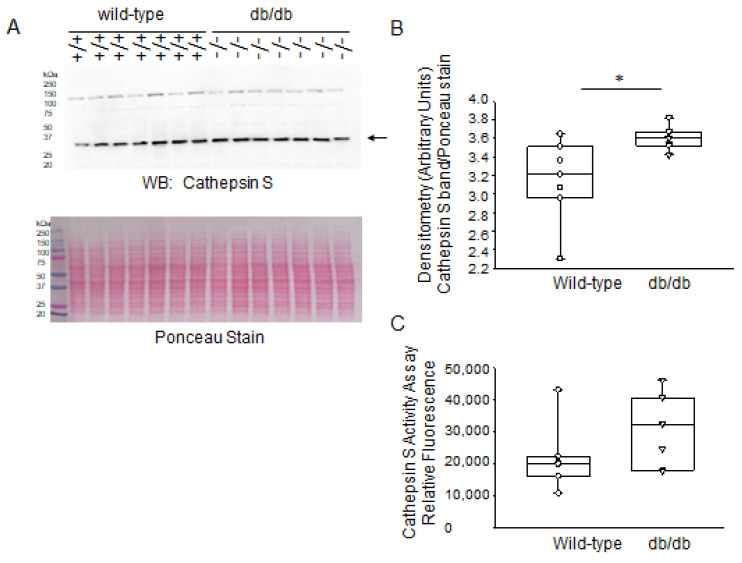
Western blot and densitometric analysis of cathepsin S protein expression in the kidneys of healthy wild-type and diabetic db/db mice. (**A**) Western blot for cathepsin S protein. Arrow indicates the immunoreactive band corresponding to cathepsin S protein. Ponceau stain was used to assess lane loading. (**B**) Densitometric analysis of the immunoreactive band in panel A indicated by an arrow. (**C**) Cathepsin S activity (shown as relative fluorescence) in kidney lysates from diabetic db/db mice compared to wild-type mice. *N* = 7 mice in each group. * represents a *p*-value of <0.05.

Next, we examined the expression of another prominent cathepsin family member in the mouse kidneys. Similar to cathepsin B and cathepsin D, Western blot and densitometric analysis showed greater basal levels of cathepsin S protein expression in the diabetic db/db kidneys compared to the kidneys of healthy wild-type mice (Figure 9A,B). The activity levels of cathepsin S was shown to be comparable between the two groups (Figure 9C).

**Figure 10:**
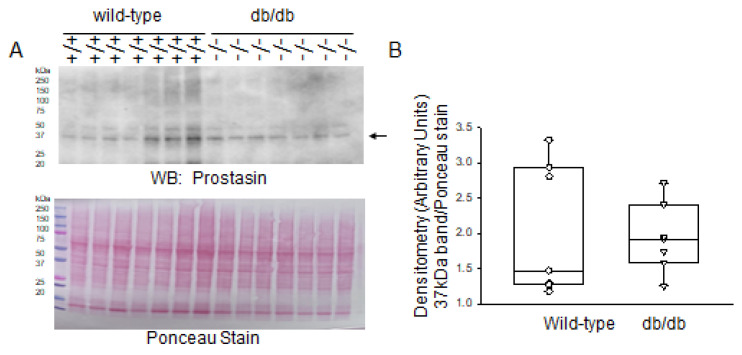
Western blot and densitometric analysis of prostasin protein expression in the kidneys of healthy wild-type and diabetic db/db mice. (**A**) Western blot for Prostasin protein. Arrow indicates the immunoreactive band corresponding to Prostasin protein. Ponceau stain was used to assess lane loading. (**B**) Densitometric analysis of the immunoreactive band in panel A indicated by an arrow. *N* = 7 mice in each group.

Next, we compared the expression of prostasin in the kidneys of diabetic db/db mice compared to healthy wild-type mice. As shown in Figure 10, the levels of prostasin were comparable between the two groups.

### 2.4. Expression Levels of Phospholipases in Diabetic db/db Kidneys Compared to Kidneys of Wild-Type Control Mice

**Figure 11:**
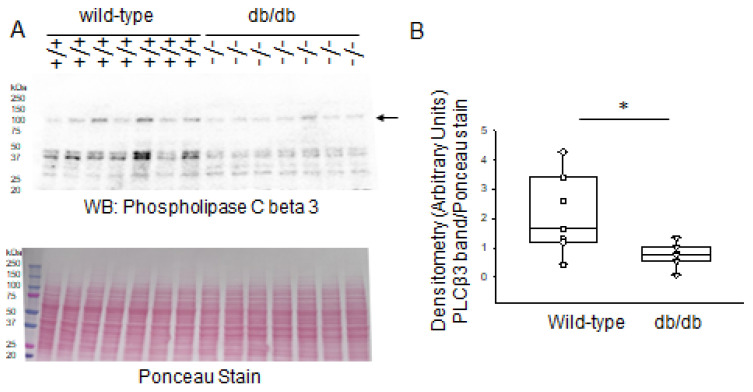
Western blot and densitometric analysis of phospholipase C beta 3 protein expression in the kidneys of healthy wild-type and diabetic db/db mice. (**A**) Western blot for phospholipase C beta 3 protein. Arrow indicates the immunoreactive band corresponding to phospholipase C beta 3 protein. Ponceau stain used to assess lane loading. (**B**) Densitometric analysis of the immunoreactive band in panel A indicated by an arrow. *N* = 7 mice in each group. * represents a *p*-value of <0.05.

In addition to PKC-mediated phosphorylation of serine residues within the effector domain of MARCKS and proteolysis of the protein by various proteases, the interaction between basic amino acids within the effector domain of MARCKS and anionic phospholipid phosphates (e.g., PIP2) within the plasma membrane plays a role in MARCKS being associated with the inner leaflet of the lipid bilayer. Therefore, we investigated changes in the expression of two different members of the phospholipase C family of proteins that are responsible for hydrolyzing PIP2 and regulating their availability at the membrane. As shown in Figure 11, basal protein expression levels of phospholipase C beta 3 were lower in the diabetic db/db kidneys compared to those of healthy wild-type littermate mice.

**Figure 12:**
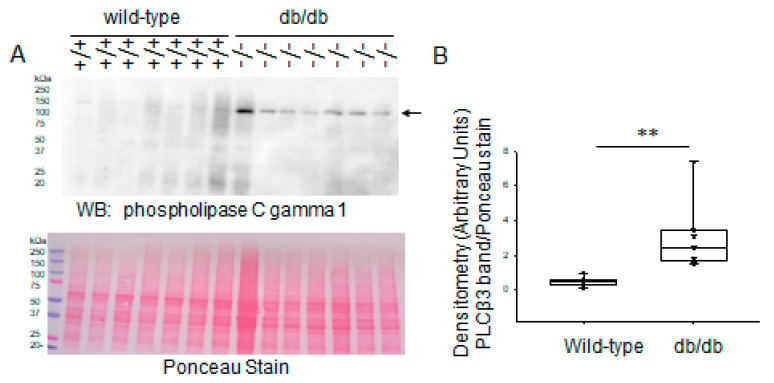
Western blot and densitometric analysis of phospholipase C gamma 1 protein expression in the kidneys of healthy wild-type and diabetic db/db mice. (**A**) Western blot for phospholipase C gamma 1 protein. Arrow indicates the immunoreactive band corresponding to phospholipase C gamma 1 protein. Ponceau stain used to assess lane loading. (**B**) Densitometric analysis of the immunoreactive band in panel A indicated by an arrow. *N* = 7 mice in each group. ** represents a *p*-value of <0.01.

Next, we investigated protein expression of phospholipase C gamma 1 in the two groups. Converse to phospholipase beta 3, basal protein expression levels of phospholipase C gamma 1 were greater in the kidneys of diabetic db/db mice compared to wild-type mice (Figure 12).

### 2.5. Expression and Proteolysis of MARCKS Protein in Mouse Cortical Collecting Duct Cells Cultured in Normal Glucose Compared to High Glucose Conditions

**Figure 13:**
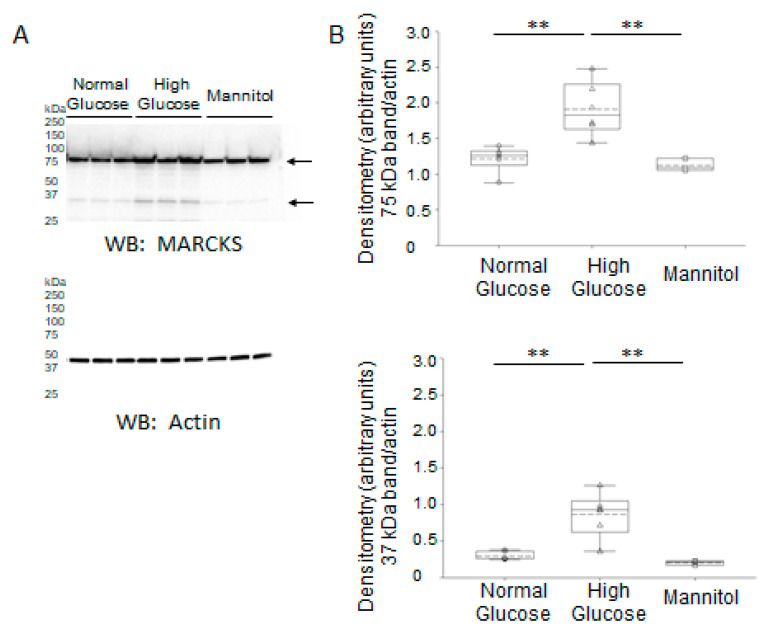
Western blot and densitometric analysis of MARCKS protein expression in mpkCCD cells. (**A**) Western blot for MARCKS protein in mpkCCD cells treated with normal glucose (5.5 mM), high glucose conditions (25 mM), or mannitol (25 mM). Western blot for actin was used to assess lane loading. Arrows indicate the immunoreactive bands for the uncleaved and cleaved forms of MARCKS protein. (**B**) Densitometric analysis of the immunoreactive bands in panel A normalized to actin. *N* = 6 independent experiments for the normal glucose and high glucose groups and *N* = 3 independent experiments for the mannitol control group. The top arrow indicates the uncleaved form (75 kDa) of MARCKS protein and the bottom arrow indicates the cleaved form (37 kDa) of MARCKS protein. ** represents a *p*-value of <0.01.

To investigate whether the increase in MARCKS expression and proteolysis could be due to high glucose, we cultured mpkCCD cells in normal and high concentrations of glucose before harvesting the cells for protein and then probing for MARCKS protein expression by Western blot and assessing the immunoreactive bands by densitometric analysis. As shown in Figure 13, MARCKS protein was augmented in mpkCCD cells cultured in high glucose conditions compared to normal glucose conditions (Figure 13). Another group of cells was treated with mannitol as an osmotic control. The expression and proteolysis of MARCKS protein were comparable in cells treated with normal glucose and mannitol (Figure 13).

**Figure 14:**
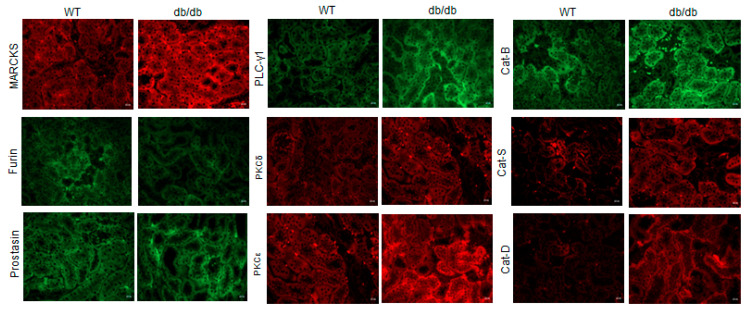
Immunohistochemistry of MARCKS, furin, prostasin, PKC and cathepsin isoforms in the diabetic and healthy mouse kidneys. *N* = 4 mice in each group. The scale bar represents 200 µm for each image. Cat B represents cathepsin B, Cat S represents cathepsin S, and Cat D represents cathepsin D.

To corroborate the changes in protein expression of MARCKS and specific proteases, kinases, and phospholipases observed by Western blot and densitometric analysis, we performed immunohistochemistry. As shown in Figure 14, MARCKS protein expression was elevated in the diabetic db/db kidneys compared to the healthy wild-type kidneys. Similarly, PLC gamma 1, PKC delta, PKC epsilon, and cathepsin B, S, and D isoforms were elevated in the diabetic db/db kidneys compared to the control group (Figure 14).

**Figure 15:**
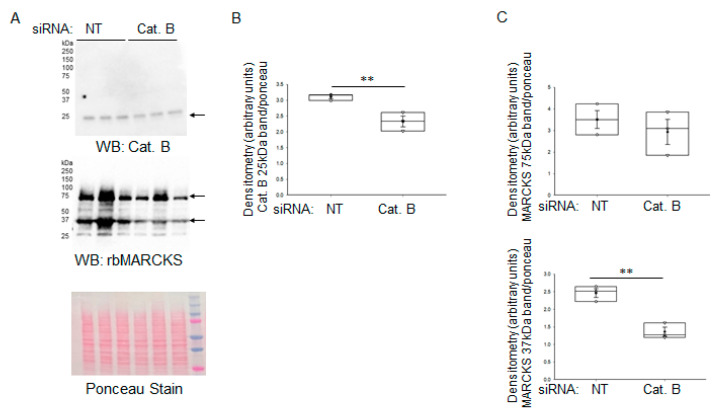
Representative Western blot of cathepsin B and MARCKS proteins after siRNA-mediated knockdown in mpkCCD cells. (**A**) Representative Western blot showing protein expression of cathepsin B (Cat. B) protein (top) and MARCKS protein (middle) from mpkCCD cells transfected with non-targeting (NT) siRNA or cathepsin B siRNA. Arrows indicate the immunoreactive bands for cathepsin B protein and MARCKS protein. Ponceau staining (bottom) was used to assess lane loading. (**B**) Densitometric analysis of the immunoreactive cathepsin B band in panel A normalized to Ponceau staining. (**C**) Densitometric analysis of the MARCKS bands in panel A normalized to Ponceau staining. *N* = 3 representing 3 independent siRNA experiments. ** represents a *p*-value of <0.01.

To further demonstrate that cathepsin B regulates MARCKS protein in the mouse collecting duct, we transfected mpkCCD cells with non-targeting (NT) control siRNA or cathepsin B specific siRNA. Western blot and densitometric analysis showed MARCKS protein was reduced in cells with an siRNA-mediated knockdown of cathepsin B. (Figure 15).

## 3. Discussion

We previously investigated renal MARCKS protein expression and proteolysis in salt-loaded hypertensive diabetic db/db mice [13]. To our knowledge this is the first study showing greater basal expression and activity of various proteases, and proteolysis of MARCKS protein, in diabetic db/db kidneys compared to the kidneys of healthy wild-type littermate mice. Here we show that a 75–80 kDa immunoreactive band corresponding to the uncleaved form of MARCKS is decreased, while a 37 kDa cleaved form of MARCKS is increased in the diabetic db/db kidneys compared to those of wild-type mice. We also show that the protein expression and activity of cathepsin B, cathepsin D, and cathepsin S are augmented in the diabetic db/db kidneys compared to those of healthy wild-type mice. These results suggest that hallmarks associated with diabetes may play a role in the upregulation of specific members of the cathepsin family of proteases and proteolysis of renal MARCKS. To investigate whether the increase in MARCKS proteolysis could at least in part be due to high glucose conditions, as in the hyperglycemic environment of the diabetic db/db kidneys, we placed mouse cortical collecting duct cells in either normal or high glucose and then measured MARCKS proteolysis by Western blotting. These experiments suggested that the cleavage of renal MARCKS may be due to high glucose conditions.

Since MARCKS is a prominent substrate of PKC, we also investigated changes in protein expression of various PKC isoforms in this study. PKC is a family of serine/threonine protein kinases that are crucial in regulating many biological processes, such as cell division, growth, and apoptosis, as well as cellular responses to environmental stressors. PKC is made up of a family of at least 12 isoforms that are divided into three groups based on how they respond to calcium and phospholipids [18]. Each PKC isoform may have distinct activities, but they have not yet been fully characterized due to the lack of specific inhibitors for each isoform and its distinct tissue distribution and subcellular localization [18]. PKC alpha, PKC delta, and PKC epsilon were reported to be activated in rat glomeruli 2 weeks after streptozotocin treatment [19]. Here, we investigated basal protein expression levels of PKC alpha, delta, and epsilon in the diabetic db/db kidneys compared to those of wild-type mice. Western blot and densitometric analyses showed that PKC delta and PKC epsilon, but not PKC alpha, were elevated in the diabetic db/db kidneys compared to the control group. The increase in protein expression of various PKC isoforms in the kidneys of diabetic db/db mice that was observed in this study is consistent with other published studies.

This is also the first study to show differential protein expression of PLC isoforms in diabetic and healthy kidneys. PIP2 plays an essential role in stabilizing ENaC at the luminal membrane through the interaction with MARCKS protein. PIP2 levels in the kidneys would presumably be elevated when PLC expression and activity are low since this enzyme hydrolyses PIP2 to yield the second messengers DAG and IP3. Although DAGs activate PKC, it is generated from either various intracellular lipid species or synthesized during de novo lipid biosynthesis of triacylglycerols and phospholipids and during catabolism of triacylglycerols stored in the endoplasmic reticulum or cytoplasmic associated lipid droplets [20]. The results presented here for the differential expression of PLCβ3 and PLCγ1 protein expression in the diabetic db/db kidneys compared to the healthy wild-type kidneys show that there is a need to further investigate the role of PLC isoforms in diabetic kidneys to better understand the interplay between PIP2 availability at the membrane and subcellular localization of MARCKS protein in diabetic kidneys.

Although this study presents novel findings on the regulation of MARCK protein in diabetic kidneys in a putative mechanism involving increased protein expression and activity of multiple cathepsins and differential protein expression of PLC isoforms, there are some limitations. First, we did not investigate whether the increased proteolysis of MARCKS by cathepsins prevents PKC mediated phosphorylation in diabetic kidneys. Our previous study suggests calpain-2 mediated cleavage of the carboxy terminal tail of MARCKS prevents its phosphorylation by PKC and translocation from the membrane [8]. Another limitation is that we did not investigate whether increased MARCKS proteolysis in diabetic db/db kidneys increases the risk of developing diabetic nephropathy. In this study we used young adult 8-week-old diabetic db/db mice, and diabetic nephropathy is typically studied several weeks later in these mice [21,22,23]. Finally, this study did not investigate how altered proteolysis of MARCKS affects the dynamics and organization of the actin cytoskeleton in the diabetic db/db kidney.

**Figure 16:**
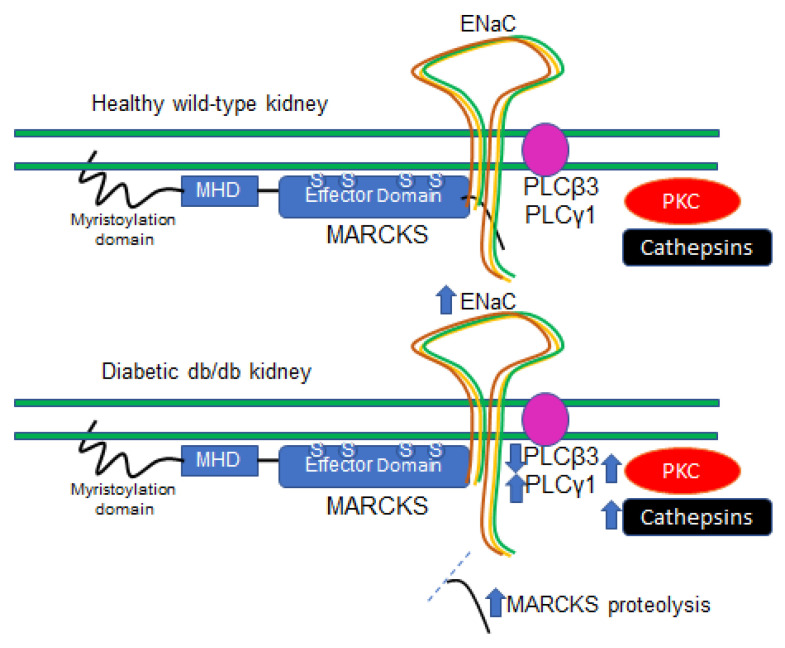
Proposed model for the regulation of MARCKS in diabetic db/db kidneys. The expression of protein kinase C (PKC) delta and epsilon isoforms is augmented in diabetic kidneys. Additionally, the expression and activity of cathepsin B, D and S isoforms are increased in diabetic kidneys. MARCKS proteolysis is greater in diabetic kidneys compared to the kidneys of healthy wild-type littermate mice. There are lower basal levels of phospholipase C β3 (PLCβ3) protein expression and greater basal levels of PLCγ1 protein expression in the kidneys of diabetic db/db mice compared to wild-type littermate mice. The proteolysis of MARCKS increases the density of the protein at the apical plasma membrane, presumably, by preventing PKC from accessing and phosphorylating serine residues within the effector domain of the protein. At the apical plasma membrane, MARCKS plays an essential role in maintaining the organization of the actin cytoskeleton and stabilizing ENaC in an open confirmation.

Collectively, these results suggest that MARCKS cleavage and association with the plasma membrane is augmented in diabetic kidneys, presumably by increased protein expression and activity of multiple cathepsin family members and decreased PLC beta 3 protein expression (Figure 16). Although high glucose appears to increase renal MARCKS proteolysis in vitro, the subcellular localization of renal MARCKS and its function at the plasma membrane warrant additional investigation.

## 4. Materials and Methods

### 4.1. Animal Studies

Seven 8-week-old male db/db mice (BKS.Cg-Dock7 m +/+ Leprdb 116/J; Stock No: 000642) and seven age-matched wild-type littermate male mice were purchased from the Jackson Laboratory (Bar Harbor, ME, USA). The mice were individually placed in metabolic cages for 24 h urine collection for one week. These animal studies were performed under an approved University of Florida Institutional Animal Care and Use Committees protocol and National Institutes of Health “Guide for the Care and Use of Laboratory Animals” guidelines.

### 4.2. Tail Bleeds for Measuring Blood Glucose

Blood glucose was measured in each group of mice using a digital glucometer (CVS Health, Woonsocket, SD, USA).

### 4.3. Measurement of Urinary Albumin

Albumin concentration was measured using an albumin assay kit (Proteintech; Rosemont, IL, USA) (Table 2) according to the manufacturer’s instructions.

### 4.4. Measurement of Urinary Creatinine

Creatinine concentration was measured using a creatinine assay kit (Abcam; Waltham, MA, USA) (Table 2) according to the manufacturer’s instructions.

### 4.5. Cathepsin Activity Assays

Cathepsin B and S activities from soluble fraction kidney cortex lysates were determined by performing cathepsin activity assays (Table 2) while following the manufactures instructions.

### 4.6. Furin Activity Assay

Furin activity in soluble fraction kidney cortex lysates was determined after performing a furin activity assay (Table 2) while following the manufactures instructions.

### 4.7. Tissue Homogenization and BCA Assay

Fifty milligrams of kidney cortex tissue were homogenized in 500 µL of tissue protein extraction reagent (TPER; Thermo Fisher Scientific, Waltham, MA, USA) using an Omni TH homogenizer (Warrenton, VA, USA) after being washed in 1× PBS (prepared from a 10× PBS solution. The tissue lysates were centrifuged at 13,000 rpm for 10 min at room temperature in a micromax benchtop centrifuge (Thermo IEC) before 450 µL of the supernatant was subject to ultracentrifugation at 34,000 rpm for 30 min at 4 °C using an optima L-90K ultracentrifuge (Beckman Coulter; Schaumburg, IL, USA) and an SW55 rotor (Beckman Coulter; Schaumburg, IL, USA). The pellets were resuspended in 200 µL TPER before being sonicated twice for 3 s at a time while on ice. A bicinchoninic acid protein assay (BCA) (Thermo Fisher Scientific) was used to determine the protein concentrations.

### 4.8. Western Blot and Densitometric Analysis

Total protein in the amount of 50µg per sample was separated on 4–20% tris HCl polyacrylamide gels at 200 V for 50 min at room temperature using a Criterion electrophoresis machine (BioRad, Hercules, CA, USA). The separated proteins were transferred onto nitrocellulose membranes (Thermo Fisher Scientific) in Towbin buffer (25 mM tris, 192 mM glycine, 20% methanol (*vol*/*vol*) using a Criterion transfer apparatus (BioRad). The membranes were blocked in 5% nonfat dry milk (prepared in 1× tris-buffered saline (TBS)) (Bio-Rad) (*w*/*v*) for 1 h at room temperature. Next, the blots were incubated in a 1:1000 dilution of primary antibody (Table 3) while rocking overnight at 4 °C. Afterwards, the membranes were rinsed three times for five minutes in 1× TBS, and then incubated with horseradish peroxidase-conjugated goat anti-rabbit secondary antibody (BioRad) (diluted 1:3000 in blocking solution) for 1 h at room temperature. The membranes were developed on an imager (Bio-Rad) after being washed four times with 1× TBS for 4 min and then incubated with ECL reagent (BioRad) for 7 min at room temperature. Image J software (IJ 1.46r) was used for the densitometric analysis.

### 4.9. Immunohistochemistry

The left kidney from each mouse was fixed in formalin for 24 h, rinsed with 1× phosphate buffered saline (PBS) (137 mM NaCl, 2.7 mM KCl, 10 mM Na_2_HPO_4_, 1.8 mM KH_2_PO_4_), placed in 70% ethanol, and then cut into 4-micrometer sections for immunohistochemistry. Paraffin-embedded kidney tissue sections were subjected to two exchanges of xylene (Fisher Scientific, Pittsburgh, PA, USA), two exchanges of 100% ethanol, one exchange of 95% ethanol, one exchange of 70% ethanol, one exchange of 50% ethanol, and finally one exchange of type-1 water for intervals of three minutes each. Next, the slides were boiled in citrate buffer (Vector Labs, Inc., Burlingame, CA, USA) for 20 min and then washed in type-1 water for three minutes. The slides were washed with 1× PBS for a period of five minutes and then blocked with normal horse serum at a concentration of 2.5% (Vector laboratories, Inc.) for 20 min in a humidified chamber. Afterwards, 200 uL of primary antibody (Table 3) was applied to each tissue section and incubated for 60 min in a humidified environment. The slides were washed three times with 1× PBS for a total of two minutes each time. After adding one drop of VectaFluor Duet Reagent (Vector labs, Inc.), the tissue was placed in a humidified chamber and allowed to incubate for 30 min at room temperature. The tissue was then washed three times for two minutes each with 1× PBS. One drop of Vectashield anti-fade mounting solution (Vector Labs, Inc.) was added and a 22 × 22-1 glass coverslip (Fisher Scientific) was applied before the slides were imaged on an Olympus BX41 microscope equipped with a 40× objective.

### 4.10. Culture and Treatment of mpkCCD Cells

Mouse mpkCCD cells were maintained in a 1:1 mixture of DMEM and Ham’s F-12 medium (GIBCO; Grand Island, NY, USA) supplemented with 20 mM HEPES, 2 mM l-glutamine, 50 nM dexamethasone, 1 nM triiodothyronine, 1× penicillin-streptomycin, and 2% fetal bovine serum (Corning-Mediatech; Woodland, CA, USA). The cells were maintained in a humidified incubation at 5% CO_2_ and at 37 °C. The amount of glucose present in the complete growth media was 5.5 mM, which was considered a normal glucose level. A separate batch of mpkCCD cell media was supplemented with additional glucose (25 mM final concentration), which was considered a high glucose level.

### 4.11. siRNA Transfection

Non-targeting control siRNA or cathepsin B (Ctsb) siRNA siGENOME SMARTpool was purchased from Horizon Discovery Biosciences (Waterbeach, Cambridge, UK), and mpkCCD cells were seeded in 6 well plates and transected with DharmaFECT reagent (Horizon Discovery Biosciences) when 70 percent confluent according to the manufacturer’s instructions.

### 4.12. Statistical Analysis

The data presented here are shown as mean values ± SEM. A Student’s *t*-test was performed to determine whether there was a statistically significant difference between the two groups. A one-way ANOVA was used to compare more than two groups. SigmaPlot software (Jandel Scientific, Corte Madera, CA, USA) was used to plot the data. A *p* value of <0.05 between the groups was considered to be statistically significant.

## Figures and Tables

**Table 1:** Characteristics between groups of mice.

Group	Wild-Type	db/db	*p*-Value
Urinary albumin (mg/dL)	0.0588 ± 0.0114	0.0669 ± 0.0253	0.77
Urinary creatinine (mg/dL)	9.187 ± 2.206	8.327 ± 1.771	0.76
Body weight (gm)	23.484 ± 0.513	34.624 ± 2.653	0.0053
Blood glucose (mg/dL)	115.143 ± 14.838	536.143 ± 12.695	8.581 × 10^−11^

**Table 2:** Sources of assays used in this study. Assay kits were purchased from Abcam (Waltham, MA, USA), BPS Bioscience (San Diego, CA, USA), and Proteintech (Rosemont, IL, USA).

Assay	Manufacturer	Catalog Number
Cathepsin B	Abcam	ab65300
Cathepsin S	Abcam	ab65307
Furin	BPS Bioscience	78040
Albumin	Proteintech	KE00076
Creatinine	Abcam	ab204537

**Table 3:** Sources of antibodies used in this study. Antibodies were purchased from Abcam (Waltham, MA, USA), Cell Signaling Technologies (Danvers, MA, USA), ThermoFisher Scientific Invitrogen, (Waltham, MA, USA), Boster Biological Technology (Pleasanton, CA, USA), and Santa Cruz Biotechnologies (Dallas, TX, USA).

Antibody	Application	Manufacturer	Catalog Number
MARCKS	WB	Abcam	ab72459
Cathepsin B	WB, IHC	Cell Signaling Technologies	31718
Cathepsin D	WB	Cell Signaling Technologies	69854
Cathepsin S	WB	Abcam	ab232740
Prostasin	WB, IHC	ThermoFisher Scientific Invitrogen	PA5-27977
Furin	WB	Cell Signaling Technologies	64709
PKC alpha	WB	Cell Signaling Technologies	2056
PKC delta	WB	Cell Signaling Technologies	9616
PKC epsilon	WB	Cell Signaling Technologies	2683
KLK1	WB	Boster Biological Technology	PA1709
PLCβ3	WB	Cell Signaling Technologies	14247
PLCγ1	WB, IHC	Cell Signaling Technologies	5690
MARCKS	IHC	Santa Cruz Biotechnologies	sc-100777
Furin	IHC	Santa Cruz Biotechnologies	sc-133142
Cathepsin S	IHC	Santa Cruz Biotechnologies	sc-271619
Cathepsin D	IHC	Santa Cruz Biotechnologies	sc-377299
PKC delta	IHC	Santa Cruz Biotechnologies	sc-8402
PKC epsilon	IHC	Santa Cruz Biotechnologies	sc-1681

## Data Availability

The individual data points from each experiment are plotted and shown within the figures of this manuscript.
